# Lifetime Prediction of Talc-Reinforced Virgin/Recycled Hybrid Polypropylene Composites Using the Arrhenius Model

**DOI:** 10.3390/ijms27188425

**Published:** 2026-09-21

**Authors:** Se-Jun Oh, Kyu-Hwan Kwon, Hui-Tae Yang, Namil Kim

**Affiliations:** 1 Department of Chemical Engineering, Hannam University, Daejeon 34054, Republic of Korea; propane01@daum.net (S.-J.O.); khkwon2100@naver.com (K.-H.K.); 2 Department of Nano-Semiconductor Engineering, Hannam University, Daejeon 34054, Republic of Korea; hty82099@naver.com

**Keywords:** recycled polypropylene, accelerated thermal aging, lifetime prediction, arrhenius model, thermal stabilization

## Abstract

The thermal durability of virgin/recycled polypropylene (PP) hybrid composites containing post-consumer recycled PP (PC-rPP) and end-of-life vehicle recycled PP (ELV-rPP) at various blending ratios was evaluated, with emphasis on the effect of a long-term thermal stabilizer (LTTS). The formulations were designed with a fixed talc content of 20 wt%, and the recycled series contained 50 wt% recycled feedstock. Accelerated thermal aging was conducted at 135, 145, and 155 °C to determine the time required to reach a 50% retention of the initial tensile strength. A common-slope Arrhenius model yielded an apparent activation energy ($E_{a,int}$) of 139.86 kJ/mol (95% Confidence Interval: 123.74–155.98 kJ/mol). Using the model-derived time to 50% retention ($t_{R50}$) at 135 °C as a reference, direct extrapolation to 100 °C yielded $t_{R50,100}$ values ranging from 5.11 to 14.04 years. At equivalent blending ratios, the rPP composites incorporating LTTS exhibited significantly extended lifetimes compared to their unstabilized counterparts. Specifically, the $t_{R50,100}\;$values for rPP composites with high ELV-rPP content (above 20 wt%) were comparable to that of the virgin PP (vPP) composite. To supplement the evaluation with absolute strength criteria, an 18 MPa tensile strength threshold was introduced. The resulting $t_{18,100}$ values spanned 2.59–9.14 years, with all recycled formulations remaining below the performance of vPP under this criterion. These values serve as conditional comparative indices because an activation energy transfer to the 18 MPa endpoint is assumed, and direct validation at 100 °C is absent. The validity of the common-slope approximation is further limited by temperature-dependent residuals and an upper-tail departure in the normal Quantile-Quantile plot.

## 1. Introduction

The automotive industry is shifting from virgin polymers to recycled alternatives in response to carbon-neutrality targets, circular-economy policies, and the increasing imperative for resource conservation [1,2]. Polypropylene (PP) is one of the most widely used plastics in automotive applications due to its low density, good processability, cost-effectiveness, and balanced mechanical performance [2,3]. In particular, talc-reinforced PP provides a balance of stiffness, dimensional stability, heat resistance, and moldability for interior trim and related components, utilizing a processing infrastructure that is already established at an industrial scale [4,5]. Replacing a portion of the virgin PP (vPP) with recycled PP (rPP) is therefore a technically viable pathway toward a circular automotive economy. However, long-term reliability remains a critical barrier to the broader adoption of rPP. Unlike vPP, rPP undergoes complex thermomechanical and oxidative degradation sustained during its prior service life and subsequent collection, post-processing, and melt reprocessing [6,7,8,9]. This cumulative exposure induces polymer chain scission, oxidative structural modifications, and alterations in crystallization, melt viscosity, and thermal stability. These molecular-level changes impair not only initial mechanical performance but also tensile strength retention and service life under prolonged thermal stress [8,9,10]. Consequently, evaluating solely short-term properties is insufficient for qualifying automotive-grade rPP composites.

Long-term durability of rPP composites is highly sensitive to the prior applications and compositional purity of the recycled sources. Specifically, end-of-life-vehicle-derived rPP (ELV-rPP), typically reclaimed from automotive components, exhibits a relatively predictable composition. In contrast, post-consumer-derived rPP (PC-rPP) is frequently recovered from mixed household or packaging waste, making it susceptible to foreign polyolefins such as polyethylene (PE), residual impurities, prior oxidation, and severe lot-to-lot variation [2,6]. Such PE contamination and subsequent matrix heterogeneity can alter the phase behavior, interfacial adhesion, and thermal stability of the PP matrix [11,12,13]. In talc-filled composites, these factors further compromise the talc–matrix interface and local stress distribution [8,14]. Although such heterogeneity may be initially masked in short-term mechanical properties, it may act as a preferential site for localized degradation, interfacial debonding, and microcrack propagation during prolonged thermal aging. This can accelerate tensile strength loss and increase uncertainty in lifetime predictions [15].

The long-term thermal stabilizers (LTTS) can delay performance loss during high-temperature exposure by scavenging radicals, decomposing hydroperoxides, and retarding oxidation [16,17,18]. Since rPP may contain pre-existing oxidized structures and degradation residues from prior uses and reprocessing, the relationship between stabilizer content and long-term thermal durability demands a more rigorous quantification than that required for vPP [19,20]. While accelerated thermal aging offers a practical approach to characterize property deterioration within a reduced experimental period, an Arrhenius analysis describes the temperature dependence of degradation rates when thermal stress acts as the dominant acceleration factor [21,22,23]. However, limited information is available on automotive talc-reinforced rPP composites that integrate the concurrent effects of ELV-rPP, PC-rPP, and LTTS. Moreover, automotive compounds based on rPP typically provide only a limited number of temperature data points per formulation, where the estimation of activation energies can introduce scatter and extrapolation uncertainty [21,22,23,24]. Therefore, a practical modeling approach is required to compare formulation-specific durability using a shared temperature-acceleration approximation.

To address these constraints, this study evaluated the long-term thermal durability of talc-reinforced virgin/recycled hybrid PP composites based on the tensile strength retention of PC-rPP/ELV-rPP blends with the talc and total recycled-feedstock contents fixed at 20 wt% and 50 wt%, respectively. Accelerated thermal aging was carried out at 135, 145, and 155 °C, where the model-derived time required for tensile strength retention to drop to 50% of its initial value was defined as $t_{R50}$. An Arrhenius model with formulation-specific intercepts and a common slope was applied to the $t_{R50}$ values of the 33 formulations, with residual-versus-fitted and normal Q–Q plots utilized to assess the model approximation. Based on the resulting $t_{R50}\;$values at 135 °C, the threshold times at 100 °C were subsequently predicted for each formulation. Furthermore, a supplementary screening assessment was conducted based on an 18 MPa screening threshold derived from GMW16456 [25]. These relative-retention and absolute-strength endpoints allow the effects of the PC-rPP/ELV-rPP ratio and LTTS to be compared at a fixed total recycled content. While the 50% retention criterion provides a clearly defined comparative endpoint with precedent in accelerated polymer-aging research, it should not be construed as a universal criterion for PP service life [26].

## 2. Results

### 2.1. Mechanical Properties Before Thermal Aging

**Figure 1:**
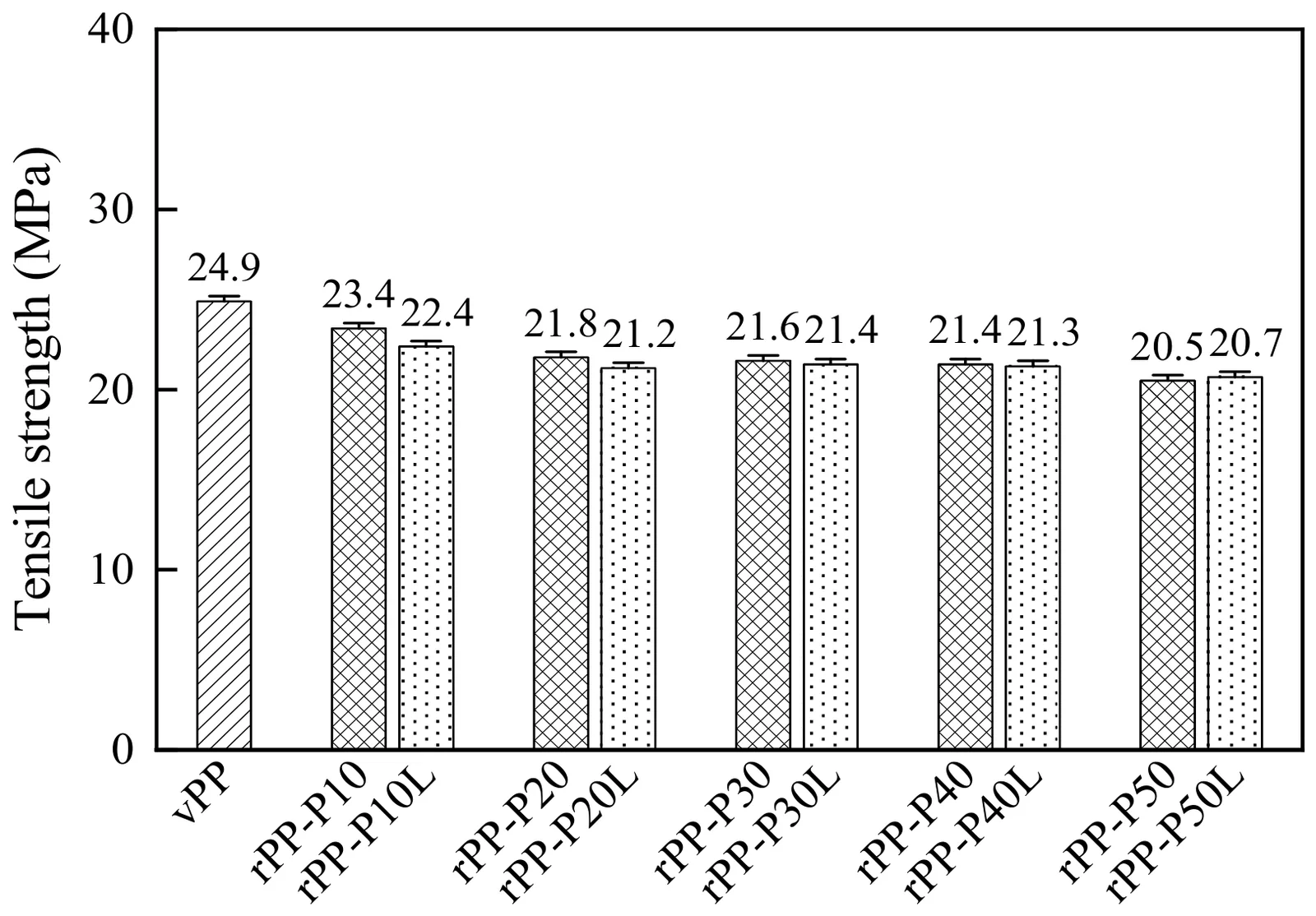
Tensile strength of the talc-reinforced PP composites for the 11 formulations before accelerated thermal aging. Error bars represent the standard deviations (SD) of five specimens, as listed in Table 1.

Table 1 summarizes the mechanical properties of the talc-reinforced virgin and recycled PP composites prior to accelerated thermal aging. Five specimens were tested for each formulation, and the results are presented with their average values. The vPP composite exhibited the highest tensile strength, flexural strength, and flexural modulus, with values of 24.9 MPa, 35.8 MPa, and 2314 MPa, respectively. Given that all formulations contained a fixed talc loading of 20 wt%, these mechanical variations are primarily attributed to the intrinsic characteristics of the vPP and rPP matrices. Among the recycled blends, rPP-P10 displayed properties closest to those of vPP, yielding a tensile strength of 23.4 MPa and a flexural strength of 33.0 MPa. The compositional dependence of the tensile strength is illustrated in Figure 1. Increasing the PC-rPP content from 10 wt% (rPP-P10) to 50 wt% (rPP-P50) gradually decreased the tensile strength to 20.5 MPa. This reduction is inferred to stem from the cumulative thermal history, potential polyethylene (PE) impurities in PC-rPP, and the resulting matrix heterogeneity within the rPP. Meanwhile, the incorporation of the thermal stabilizer exerted a negligible effect on the initial tensile and flexural properties due to its low content. For instance, the tensile strengths of the stabilized composites (rPP-P10L to rPP-P50L) varied by less than 1 MPa compared to their unstabilized counterparts. This confirms that the low stabilizer loading did not adversely affect the initial mechanical performance.

In contrast, the impact strength of the rPP composites, ranging from 4.9 to 6.9 kJ/m^2^, was higher than that of vPP (4.4 kJ/m^2^). This strength–toughness trade-off can be attributed to the heterogeneous nature of the rPP matrix. As confirmed by DSC analysis, the rPP composites displayed dual melting peaks at approximately 125 °C and 164 °C, whereas vPP exhibited a single sharp peak at 165 °C (Table 1). The heat of fusion (∆Hm) associated with the lower melting peak increased with increasing PC-rPP fractions, indicating the presence of a PE-rich secondary phase within the rPP matrix [11,12,13].

### 2.2. Estimation of Threshold Times from Tensile Strength Retention

**Figure 2:**
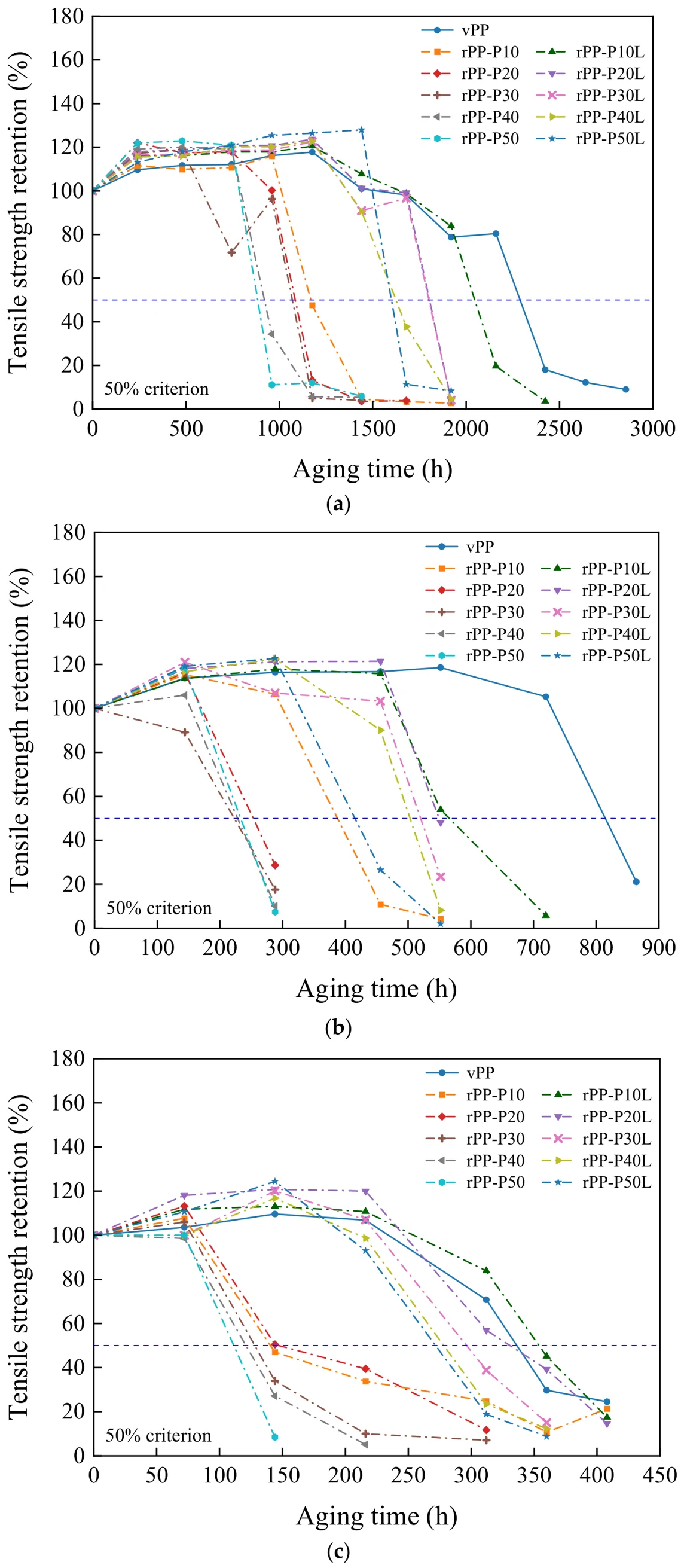
Tensile strength retention during accelerated thermal aging at (**a**) 135 °C, (**b**) 145 °C, and (**c**) 155 °C. Chronological variations, including values exceeding 100% and localized rises, are plotted. The 0 h reference (100%) is based on the mean initial tensile strength of five replicate specimens (*n* = 5); each post-aging point represents a single measurement (*n* = 1).The horizontal dashed line denotes the 50% tensile strength retention criterion used to determine tR50.

Figure 2 shows the tensile strength retention behavior as a function of aging time at 135, 145, and 155 °C. The retention profiles displayed an initial increase above 100% and subsequent local fluctuations in certain formulations. Overall, these curves exhibited a progressive long-term decline. The model-derived time required to reach the 50% retention threshold ($t_{R50}$) decreased with increasing aging temperature. However, the absolute profiles and the onset of rapid degradation varied depending on the formulation. As shown in Figure 2a, vPP showed the longest $t_{R50}$ among the unstabilized formulations. Furthermore, the stabilizer-containing formulations demonstrated prolonged periods of high strength retention compared to their unstabilized counterparts. When the aging temperature increased to 145 °C, the divergence among the formulations became more pronounced within a narrower time frame, although local rises and experimental scatter still persisted (Figure 2b). At 155 °C, the unstabilized composites with higher PC-rPP fractions rapidly dropped below the 50% retention level, with rPP-P50 demonstrating the most rapid decline (Figure 2c). The extended retention times of the LTTS-containing formulations are consistent with the stabilizing effects discussed in prior PP aging studies [16,17,20], although oxidation chemistry and chain scission were not directly monitored in this work.

**Figure 3:**
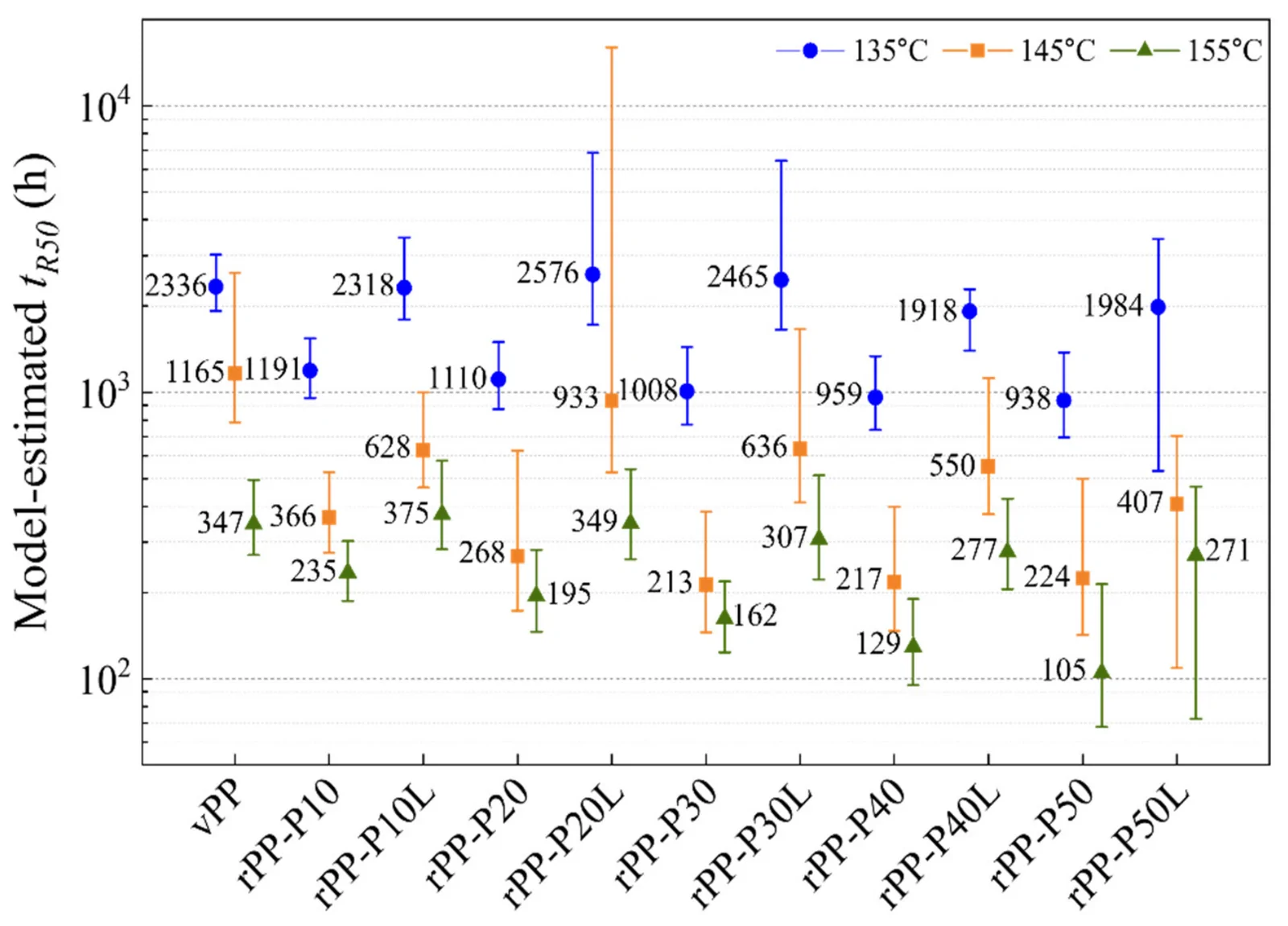
Model-derived $t_{R50}$ estimates and two-sided 95% model-based inverse-prediction confidence intervals at 135, 145, and 155 °C. The time axis is logarithmic; error bars represent confidence intervals for the fitted model’s time to 50% retention.

The model-estimated threshold times ($t_{R50}$) for each formulation at 135, 145, and 155 °C, obtained via the inverse-prediction procedure described in Section 4.4, are displayed in Figure 3 with 95% confidence intervals on a logarithmic time scale. For vPP, the $t_{R50}$ values decreased from 2336 h at 135 °C to 1165 h at 145 °C, and further to 347 h at 155 °C. In contrast, the unstabilized rPP-P50 formulation, which exhibited the poorest thermal resistance, declined from 938 h at 135 °C to 224 h at 145 °C and 105 h at 155 °C. Although its stabilized counterpart, rPP-P50L, also exhibited a reduction from 1984 h (135 °C) to 271 h (155 °C), it maintained longer threshold times than rPP-P50 across the entire temperature range. The effect of the long-term thermal stabilizer (LTTS) was evaluated at identical blending ratios. At 135 °C, the $t_{R50}$ values for the stabilized series from rPP-P10L to rPP-P50L were 2318, 2576, 2465, 1918, and 1984 h, respectively, consistently exceeding those of their respective unstabilized counterparts.

### 2.3. Common-Slope Arrhenius Analysis

**Figure 4:**
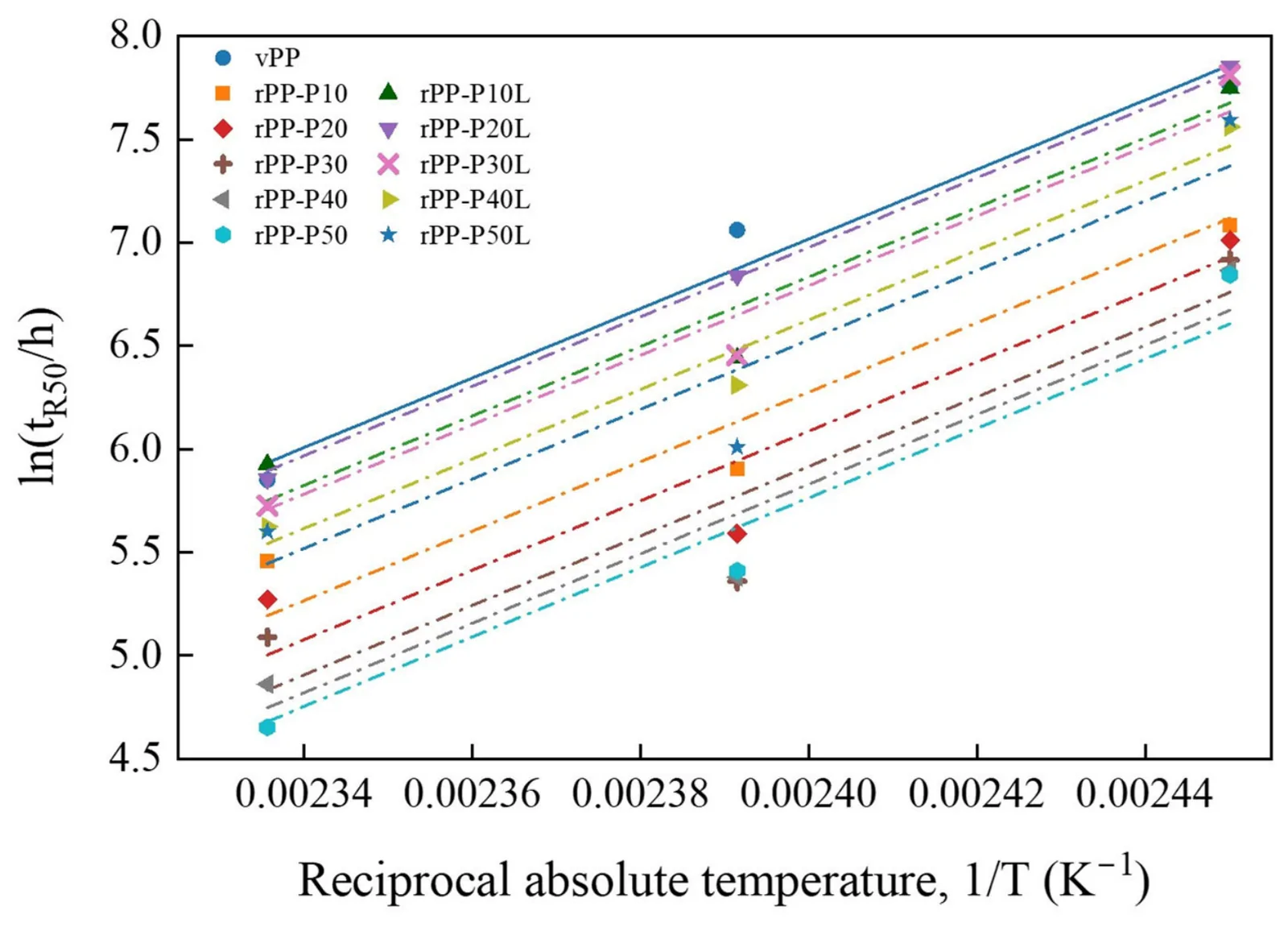
Common-slope Arrhenius plot of 33 model-derived tR50 values against reciprocal absolute temperature, with formulation-specific intercepts and a shared temperature slope.

Figure 4 presents the Arrhenius plot of the model-derived $t_{R50}$ values against reciprocal absolute temperature (1/T). An Arrhenius model utilizing a formulation-specific intercept and a common-slope was applied. The interaction test between formulation and 1/T yielded F(10,11)=0.1179 and p=0.9989. Because each formulation was evaluated at only three temperatures, this statistical outcome does not establish identical activation energies. Nevertheless, the absence of a detectable slope difference, combined with the advantage of a reduced parameter count, motivated the selection of the common-slope model as a comparative approximation. The estimated common reciprocal-temperature slope was 16,821.303 K, which corresponds to an apparent activation energy ($E_{a,int}$) of 139.86 kJ/mol (95% CI: 123.74–155.98 kJ/mol). The common-slope regression yielded an $R^{2}=0.9535$, adjusted $R^{2}=0.9291$, and RMSE = 0.2503 on the ln($t_{R50}$) scale, with model and error degrees of freedom were 11 and 21, respectively. Although incorporating the interaction term slightly increased $R^{2}$ to 0.9580, the adjusted $R^{2}$ decreased to 0.8778 and the RMSE increased to 0.3287. This comparison supports a parsimonious approximation for the three-temperature experimental design, rather than serving as a direct validation of low-temperature extrapolation. 

**Figure 5:**
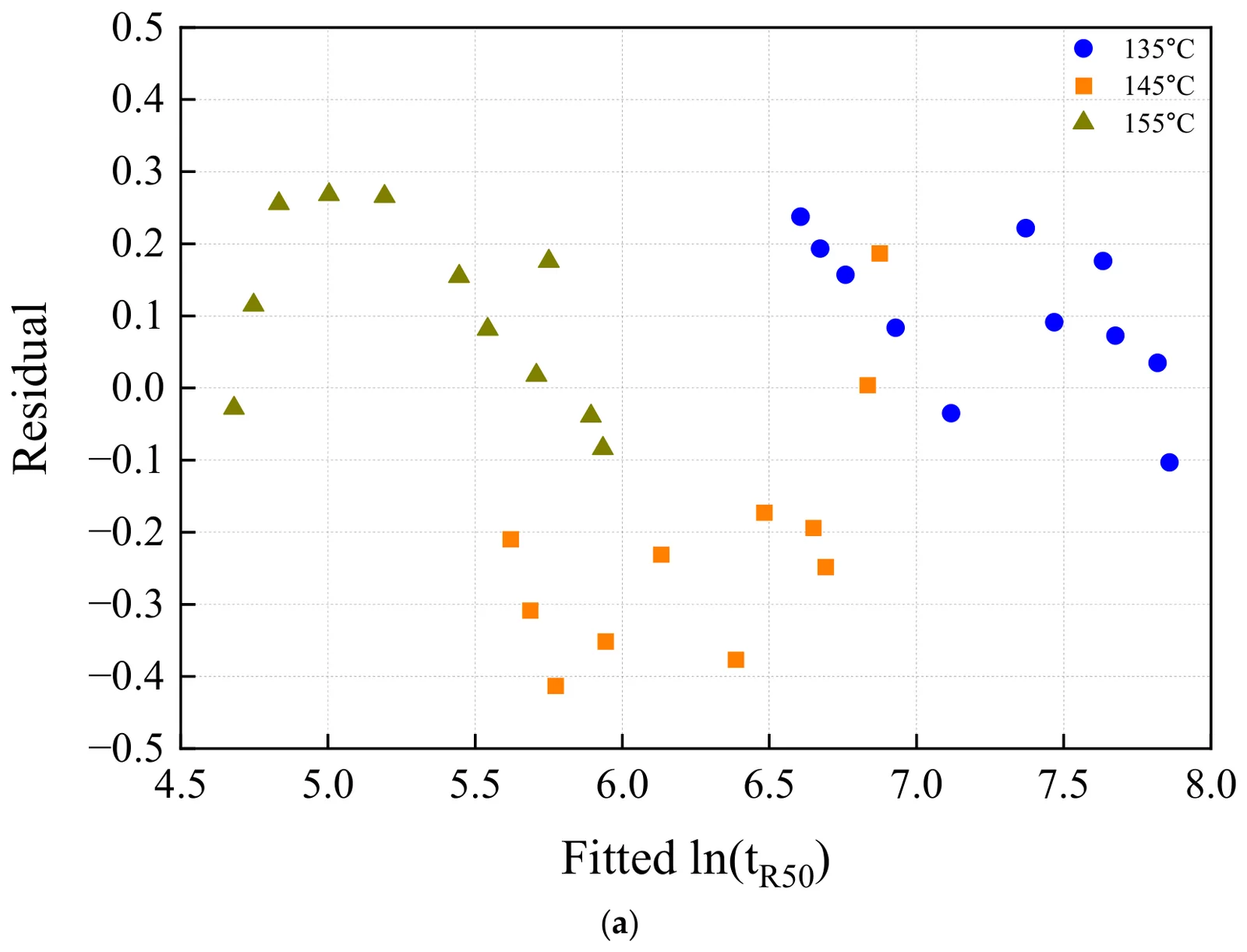

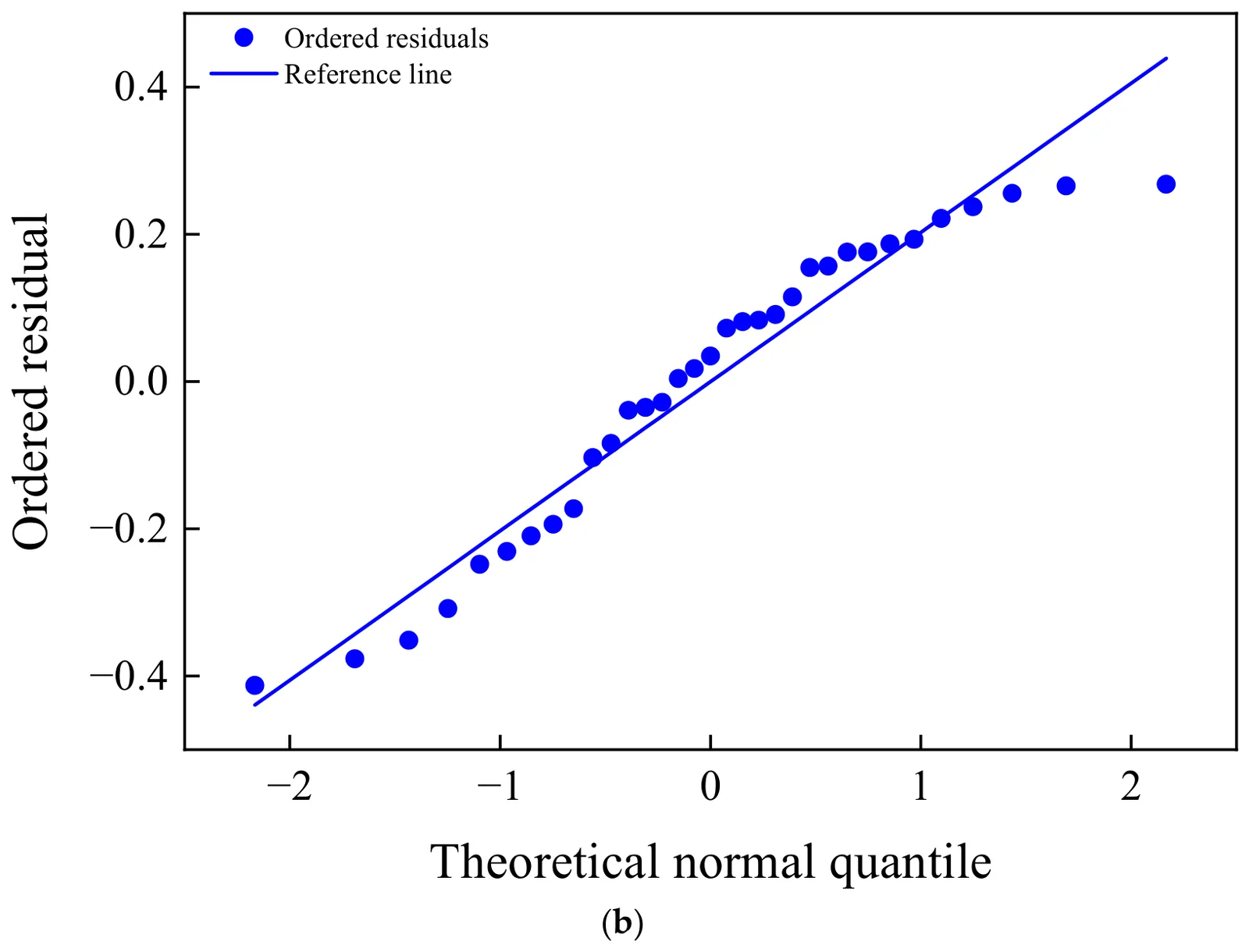
Residual diagnostics for the common-slope Arrhenius regression of 33 model-derived $t_{R50}$ values at 135 °C, 145 °C, and 155 °C. (**a**) Ordinary residuals versus fitted values, with a zero reference line. (**b**) Normal Q–Q plot of the same residuals, with a reference line.

Figure 5 shows the residual diagnostics for the common-slope Arrhenius regression of the 33 model-derived $t_{R50}$ values. Ordinary residuals were defined as the input $ln\left(t_{R50}\right)$ minus the fitted value. The mean residual at 145 °C was −0.2102, with 9 of the 11 residuals exhibiting negative values (Figure 5a). The mean residuals at 135 and 155 °C were +0.1026 and +0.1076, respectively. These results indicate that the fitted values generally exceeded the inputs at 145 °C, while being lower on average at the two endpoint temperatures. Furthermore, in the upper tail of the normal Q–Q plot, the largest positive residuals departed below the theoretical reference line (Figure 5b). These findings suggest that the residuals do not strictly conform to a random distribution or absolute normality; consequently, the error structure remains a limitation of the model despite its overall fit.

### 2.4. Lifetime Prediction of rPP Composites via 135 °C Anchor-Based Extrapolation

**Figure 6:**
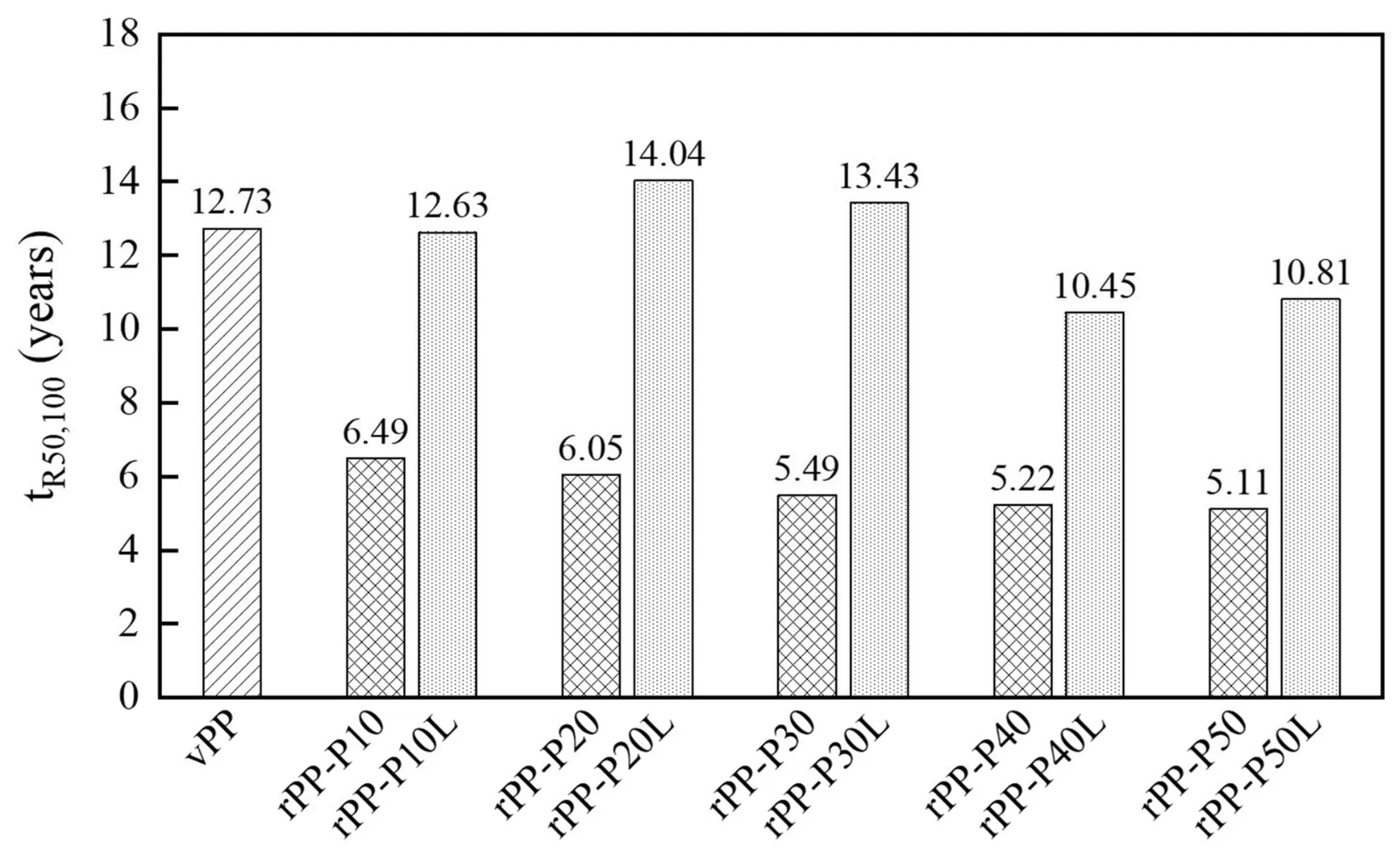
Model-extrapolated times to 50% initial tensile strength retention at 100 °C (tR50,100) for the 11 talc-reinforced PP formulations. Each value is a point estimate obtained by applying the common Arrhenius acceleration factor (AF135 → 100) to the formulation-specific model-derived tR50 at 135 °C. The values are not independent-specimen failure probabilities, product warranty lives, or formal material-qualification results.

A 50% tensile strength retention was utilized as the primary criterion to evaluate the relative degradation of the formulations under accelerated aging. While this endpoint accounts for strength differences, it is distinct from a universal PP service life limit. Using the composition-dependent $t_{R50}$ values obtained at 135 °C as a baseline, the threshold times at 100 °C were predicted by applying the common Arrhenius acceleration factor (AF135→100 = 47.7351) and converting hours to years based on a conversion factor of 8760 h/year. Figure 6 illustrates the predicted times to 50% retention ($t_{R50,100}$), ranging from 5.11 years for rPP-P50 to 14.04 years for rPP-P20L. For comparison, vPP, rPP-P10L, and rPP-P30L yielded $t_{R50,100}$ values of 12.73, 12.63, and 13.43 years, respectively. At identical PC-rPP/ELV-rPP ratios, the incorporation of the thermal stabilizer increased the $t_{R50,100}$ by 94.6% for rPP-P10, 132.0% for rPP-P20, 144.6% for rPP-P30, 100.1% for rPP-P40, and 111.5% for rPP-P50. Because applying a uniform acceleration factor mathematically preserves the ranking and proportions of the 135 °C reference values, these comparisons do not experimentally establish the actual lifetime ranking at 100 °C.

**Figure 7:**
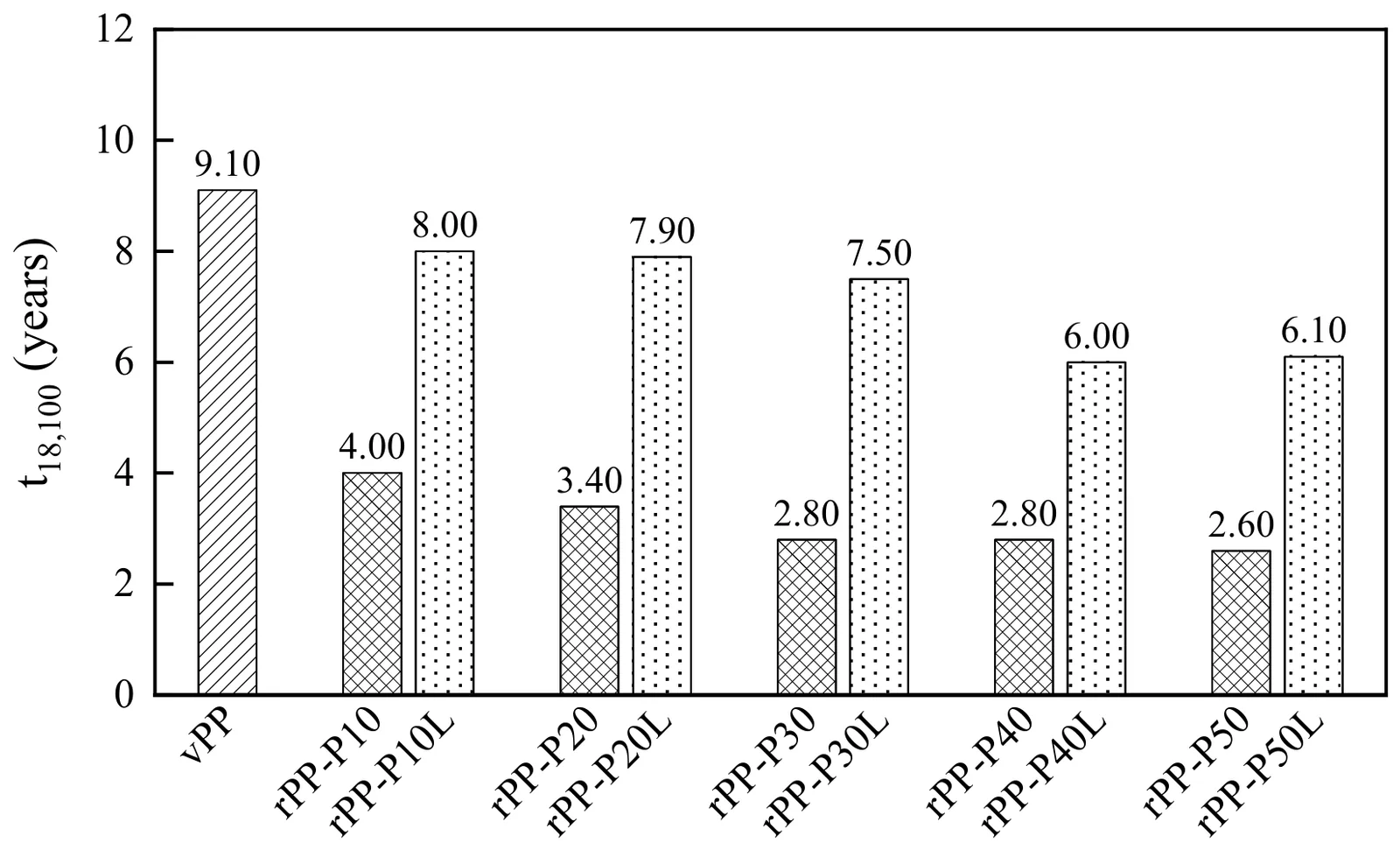
Conditional model-extrapolated times to a fixed tensile strength of 18 MPa at 100 °C (t18,100) for the 11 talc-reinforced PP formulations. Each value is calculated from the formulation-specific model-derived t18 at 135 °C using the common Arrhenius acceleration factor based on Ea,int from the primary tR50 analysis. Transfer of activation energy between endpoints is assumed. The 18 MPa threshold, derived from 24 MPa × 75%, is a research screen and does not represent formal GMW conformity, failure probability, or product warranty life.

For an absolute-strength comparison, an 18 MPa threshold was introduced as a reference value, calculated from the 24 MPa initial strength requirement and 75% retention after 1000 h at 100 °C according to GMW16456 for the 20 wt% talc-reinforced class [25]. This fixed threshold was applied uniformly to every formulation and does not represent 75% of each individual formulation’s specific initial strength. The model-derived $t_{18}$ values at 135 °C were multiplied by the same acceleration factor (AF135 → 100) used for the $t_{R50}$ evaluation. This approach implicitly assumes the transfer of the apparent activation energy ($E_{a,int}$) to the 18 MPa absolute endpoint, as no separate $E_{a,18}$ fitting process was performed. As presented in Figure 7, vPP exhibited the longest $t_{18,100}$ value at 9.14 years. The stabilized rPP-P10L, rPP-P20L, rPP-P30L, rPP-P40L, and rPP-P50L yielded values of 7.97, 7.87, 7.50, 5.98, and 6.07 years, respectively. While these values remained below that of the vPP, they represented a 98.2–171.4% increase over their corresponding unstabilized counterparts. This 18 MPa result serves as a research-screening index for formulation comparison, rather than a formal GMW16456 conformity determination.

## 3. Discussion

Thermal durability was compared using model-extrapolated lifetimes to defined tensile strength thresholds. Under the 50% retention criterion, the $t_{R50,100}$ value of rPP-P10L was comparable to that of vPP, while rPP-P20L and rPP-P30L surpassed the vPP. Under the absolute 18 MPa criterion, the stabilized rPP formulations remained below the performance of vPP across all compositions. While the 50% retention criterion scales proportionally with specific initial strength depending on formulation, the fixed 18 MPa threshold is reached at a higher relative retention level in recycled formulations possessing lower initial strength. Because these endpoints embody fundamentally different physical meanings, a ranking under one criterion does not establish overall equivalence or absolute superiority in long-term thermal durability. Nevertheless, the extended threshold times of every stabilized formulation relative to its unstabilized counterpart under both criteria provide a valid, conditional basis for screening recycled feedstock blends and optimizing stabilization conditions.

Comparison with the commercial-grade vPP reference, which has a history of meeting the applicable automotive specification, provides a practical benchmark for recycled-PP formulations. At 50 wt% total recycled feedstock, the $t_{18,100}$ estimates of rPP-P10L and rPP-P20L were 7.97 and 7.87 years, respectively, compared with 9.14 years for vPP. For rPP-P50L containing 50 wt% PC-rPP, the $t_{18,100}$ was 6.07 years, compared with 2.59 years for the unstabilized rPP-P50. These results motivate further evaluation of recycled content, PC-rPP/ELV-rPP ratio, and LTTS conditions. Reducing the total recycled content was not evaluated in this study, and whether such modifications can approach vPP-level durability remains a hypothesis requiring subsequent formulation trials and direct aging verification at 100 °C. The specification history of reference material does not make the present 18 MPa extrapolation a new conformity assessment.

The aging temperatures of 135–155 °C are located below the melting temperature of PP near 164–165 °C but remain above the secondary melting transition near 125 °C. Consequently, extrapolation of the experimental results to a service temperature of 100 °C crosses this transition and may be subject to non-Arrhenius behavior, alterations in the dominant degradation mechanism, and oxygen diffusion limitations [21,23,24]. The present $E_{a,int}$ is an apparent value based on tensile strength retention and should not be directly equated with activation energies obtained from mass loss or thermogravimetric analysis (TGA). The projected 100 °C values must be interpreted as comparative indices conditional on the high-temperature dataset and the common-slope assumption. Therefore, direct lower-temperature validation remains necessary. The activation energies of PP depend on the response and experimental analysis conditions. For instance, Esmizadeh et al. reported activation energies of approximately 70–160 kJ/mol during oxidative degradation in non-isothermal TGA [10], whereas the comprehensive review by Celina et al. discussed values of approximately 107–156 kJ/mol for high-temperature PP processes and 35–50 kJ/mol for lower-temperature processes [23]. While the current value of 139.86 kJ/mol is compatible with the reported higher-temperature range, numerical agreement across distinct materials and response metrics does not conclusively establish an identical degradation mechanism or validate low-temperature extrapolation. The temperature-dependent residual pattern in Figure 5 shows that the common-slope regression does not fully describe the variation across the three temperatures. It does not identify a particular degradation mechanism or cause of curvature, but it limits the single apparent activation energy approximation. Moreover, the diagnostic inputs are 33 model-derived crossing times. These diagnostics do not directly assess tensile-test repeatability, dependence and uncertainty among crossing estimates, or the accuracy of the 100 °C extrapolation.

**Figure 8:**
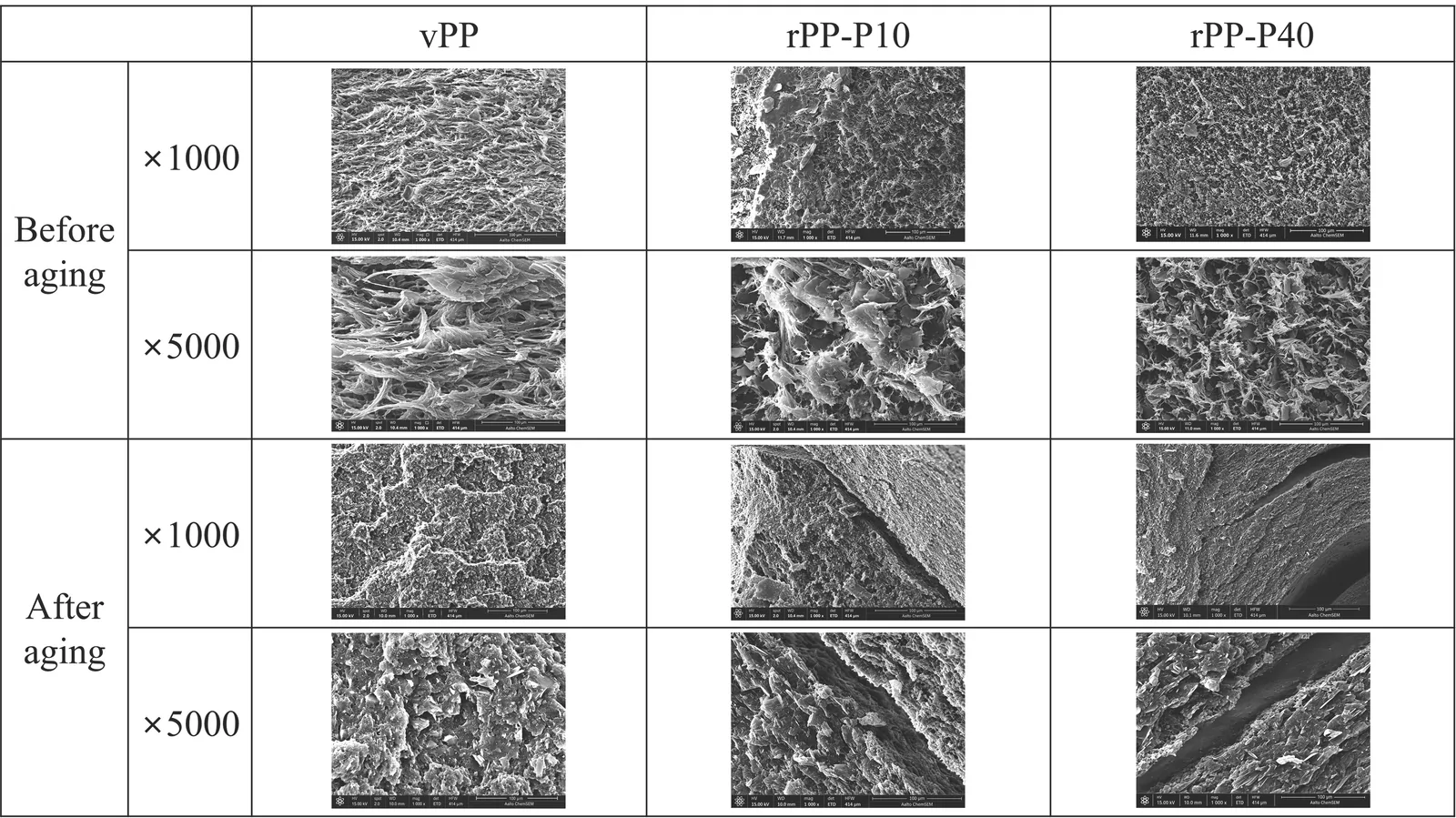
SEM fracture-surface images of vPP, rPP-P10, and rPP-P40 before and after thermal aging at ×1000 and ×5000 magnification. “Before aging” denotes 0 h before oven exposure. “After aging” denotes the formulation-specific final time at 135 °C: 2856 h for vPP, 1920 h for rPP-P10, and 1440 h for rPP-P40.

Figure 8 presents the SEM micrographs of the fracture surfaces of vPP, rPP-P10, and rPP-P40 taken before and after thermal aging at 135 °C, where rPP-P10 and rPP-P40 were selected to investigate the effect of PC-rPP content on morphological changes. A similar morphology is expected for rPP-P50 considering its tensile strength retention behavior. Prior to aging, no discernible structural differences or defects were observed across the formulations. However, after thermal aging, surface cracks were clearly identifiable in rPP-P10 and rPP-P40 even upon visual inspection. Such microcracking was less pronounced in the vPP. These morphological differences are qualitatively consistent with the observed tensile strength degradation profiles.

**Figure 9:**
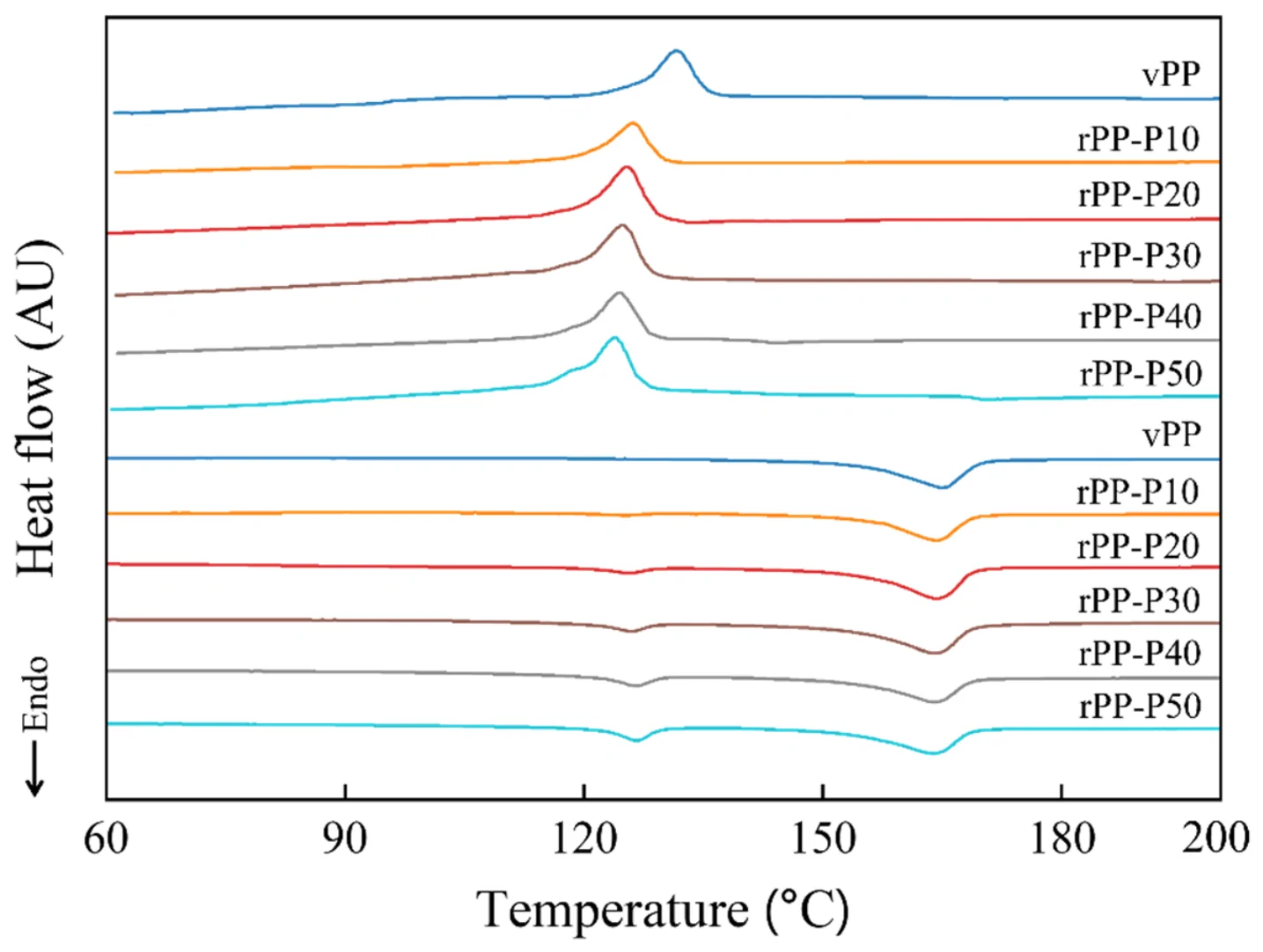
DSC thermograms of vPP and the unstabilized rPP formulations (rPP-P10 to rPP-P50) during heating and cooling. Curves were reconstructed by digitizing the measurement-output graphs.

Figure 9 displays the DSC thermograms of the vPP and rPP series (rPP-P10 to rPP-P50) obtained during the heating and cooling cycles. The vPP exhibits a prominent single melting peak at approximately 165 °C and a crystallization peak at 131 °C. In contrast, the recycled formulations exhibit dual melting peaks, comprising a major endothermic peak at 164 °C and a minor peak at 125 °C. Notably, the intensity of this low-temperature peak becomes more pronounced as the fraction of PC-rPP increases from rPP-P10 to rPP-P50. The presence of this secondary peak at around 125 °C, along with its compositional intensity variations, is consistent with the presence of a PE-rich secondary phase in post-consumer feedstocks (PC-rPP), thereby providing a plausible morphological context for the observed strength–toughness balance. DSC peaks alone do not identify the PE type, quantify its content, or establish PE as the sole cause of the mechanical trends.

## 4. Materials and Methods

### 4.1. Materials and Formulation Design

Two types of recycled polypropylene—end-of-life vehicle-derived recycled PP (ELV-rPP, Yongsan Chemical Co., Ltd., Gimje, Republic of Korea), and post-consumer recycled PP (PC-rPP, GPINP-310K, SM Tech Co., Ltd., Pyeongtaek Republic of Korea)—were employed to evaluate initial mechanical performance, accelerated thermal aging behavior, and tensile strength-based lifetime.Virgin polypropylene (vPP, HA5029, PolyMirae Co., Ltd., Seoul, Republic of Korea) served as a reference. Platelet-like talc (KC-2000, KOCH Co., Ltd., Seocheon, Republic of Korea) was added at a fixed 20 wt% to improve matrix stiffness, dimensional stability, and thermal deformation resistance.The hybrid virgin/recycled PP blends contained 50 wt% recycled feedstock at varying PC-rPP/ELV-rPP ratios, and 0.3 wt% long-term thermal stabilizer (LTTS, RECYCLOBYK 4371, BYK-Chemie GmbH, Wesel, Germany) was incorporated where applicable.In addtion, JMP Student Edition, version 19.1.3 (SAS Institute Inc., Cary, NC, USA) was used to predict the lifetime.Table 2 outlines the sample designations and compositions. The recycled-series formulations are hybrid matrices: the unstabilized formulations contain 30 wt% vPP, 50 wt% recycled PP feedstock, and 20 wt% talc, while the LTTS-containing formulations contain 29.7 wt% vPP, 50 wt% recycled PP feedstock, 20 wt% talc, and 0.3 wt% LTTS. Holding the virgin fraction fixed within each series provided a controlled comparison of the recycled-feedstock ratio and LTTS addition.

### 4.2. Melt Processing and Accelerated Thermal Aging

Specimens for each formulation were produced from the same manufacturing lot. All components were melt compounded using a co-rotating twin-screw extruder with an L/D ratio of 40:1 (JTE-32M, Jireh Tech, Siheung, Republic of Korea). The screw configuration incorporated conveying, distributive and dispersive mixing elements, including three kneading blocks with nominal disk angles of 45°, 60° and 90°, alongside a venting region located at approximately L/D 28–32. To minimize processing-induced variations, a uniform feeding sequence, screw design, and process window were maintained across all formulations. Subsequently, injection molding was performed using an injection molding machine (SELEX E150, Woojin Plaimm, Boeun, Republic of Korea). The actual extrusion settings for barrel zones 1–9 were 180, 185, 190, 195, 200, 205, 210, 210, and 220 °C, respectively; adapter and die temperatures were 210 and 220 °C. The screen pack was 40/60 mesh, the screw speed was 350 rpm, the pelletizer speed was 260 rpm, and the throughput was 30 kg/h. Materials were dried at 90 °C for 4 h before injection molding. The actual injection-mold parameters were established as follows: barrel temperature of 210–220 °C across the rear, middle, and front zones; nozzle temperature of 220 °C; a mold temperature of 60 °C; an injection pressure of 80 MPa; a back pressure of 0.5 MPa; a screw speed of 80 rpm; and a cooling time of 30 s. Specimens with incomplete filling, visible bubbles, severe warpage, burn marks, or surface cracks were discarded.

Accelerated thermal aging was carried out at three different temperatures at 10 °C intervals (135, 145, and 155 °C) to induce distinct temperature-dependent degradation rates and provide the kinetic data required for the subsequent integrated Arrhenius analysis. The specimens were placed in an internal air-circulating oven with sufficient spacing to avoid mutual contact and reduce shielding. For each formulation, tensile strength was measured at 0 h and at formulation- and temperature-specific aging times.After aging, specimens were removed from the oven, cooled and conditioned at room temperature for 24 h. The 135 °C condition provided the baseline degradation history for each formulation in the final lifetime prediction.

### 4.3. Mechanical Testing and Characterization

The mechanical properties were evaluated via tensile (ISO 527-2, Type 1BA) [27], flexural (ISO 178) [28], and notched Charpy impact (ISO 179-1) testing [29] to investigate the effects of the compounding design. Tensile and flexural properties were measured using a universal testing machine (AG-X Plus, Shimadzu Corporation, Kyoto, Japan), while impact strength was determined using an impact tester (QM700A, QMESYS Corp., Uiwang-si, Republic of Korea). Tensile strength testing was conducted at a crosshead speed of 50 mm/min for both initial and aged specimens, while tensile modulus testing was performed at 1 mm/min, flexural testing at 2 mm/min, and Charpy impact testing at 23 °C. Tensile strength was defined as the maximum tensile stress recorded during the test, distinct from the stress at fracture after the maximum, and was the primary response used for threshold-time and Arrhenius analyses. Initial mechanical properties are reported as the mean ± sample SD for five specimens per property/test condition (n = 5). Each recorded post-aging tensile value is a single measurement for a formulation/temperature/time point (*n* = 1), so no replicate mean or SD is reported for aged points and the initial-property SD was not applied to them. Tensile strength retention for formulation i at temperature T and time t was calculated using Equation (1).

(1) $$R_{i,T}\left(t\right)=\frac{\sigma_{i,T}\left(t\right)}{\sigma_{i,T}\left(t=0\right)}\times 100$$

where $\sigma_{i,T}\left(t\right)$ represents the tensile strength measured after aging at temperature T for time t. The denominator is the mean initial tensile strength for each formulation recorded to one decimal place in MPa, and the retention at 0 h was set to 100%.

Differential scanning calorimetry (DSC) (DSC 4000, Perkin Elmer, CT, USA) was employed to measure the melting temperature (Tm) and the corresponding heat of fusion (∆Hm) of the rPP blends [30]. Each composite weighing around 3~5 mg was sealed in an aluminum hermetic cell and scanned at a constant heating/cooling rate (10 °C/min). During the heating/cooling process, the chamber was maintained under a nitrogen atmosphere (N_2_) at a flow rate of 50 mL/min to maintain an inert atmosphere. Scanning electron microscopy (SEM) (PHENOM-XL, ThermoFisher, Waltham, MA, USA) analysis was conducted to examine matrix continuity and microstructural features, such as void and microcrack formation, as well as talc pull-out after thermal aging. The specimens were sputtered with silver for 90 s using a sputtering device before characterization. An acceleration voltage of 15 kV was employed. Micrographs were captured that corresponded to the cross-sectional and surface morphology. These morphological observations provided qualitative information for interpreting the reduction in tensile strength retention.

### 4.4. Data Preprocessing and Lifetime Prediction Methodology

Lifetime prediction was conducted using model-estimated threshold times derived from tensile strength retention. The primary objective of this data processing was to quantify retention loss as a function of aging time rather than merely comparing the initial tensile strength across formulations. Retention values exceeding 100% during the early aging stage were retained, as they can arise from measurement scatter, transient structural stabilization upon thermal exposure, or test variability. No data point was excluded solely because it was inconsistent with the overall degradation trend. This dataset was subsequently used to estimate $t_{R50,T,i}$ defined as the fitted time required to reach 50% tensile strength retention for formulation i at aging temperature T. Final threshold times at 100 °C were calculated directly from the formulation-specific 135 °C anchors using the common Arrhenius acceleration factor.

The $t_{R50}$ values were obtained in JMP’s Destructive Degradation platform, with tensile strength retention (%) as the response, aging time (h) as the time variable, temperature (°C) as the explanatory variable, analysis by formulation, and a lower specification limit of 50%. To report both fit and crossing-time uncertainty, the model with the lowest AICc was selected for each formulation among the evaluated candidates capable of producing a 95% inverse-prediction confidence interval. JMP (JMP Student Edition, version 19.1.3, SAS Institute Inc., Cary, NC, USA).inverse prediction used Confidence Interval, α = 0.05, and Two-sided settings to obtain two-sided 95% model-based inverse-prediction confidence intervals for the fitted time to 50% retention. A Simple Linear model with linear X/Y scales and a Normal location-parameter path was used; no separate monotonicity constraint was imposed. Within each formulation, the model used a common intercept and temperature-specific slopes, and the time at which fitted retention reached 50% was obtained by inverse prediction. The fitted linear path has one 50% crossing, so no crossing was manually selected from multiple raw-data crossings. Six values are fitted-model extrapolations beyond the observed range: vPP at 145 °C; rPP-P20L at 135 and 145 °C; rPP-P30L at 135 and 145 °C; and rPP-P50L at 135 °C. The $t_{R50}$ values and confidence intervals are therefore inverse predictions conditional on the specified fitted model.

For the Arrhenius analysis, the $t_{R50}$ datasets obtained at 135, 145, and 155 °C were consolidated into a single pooled dataset. The Arrhenius regression was linearly modeled between ln(tR50/h) and reciprocal absolute temperature (1/T), as shown in Equation (2).

(2) $$\ln(\frac{t_{R50,i,T}}{h})=a_{i}+\frac{b}{T}+\varepsilon_{i,T}$$

Here, ai is the formulation-specific intercept, b is the common reciprocal-temperature slope, T the absolute temperature in Kelvin, and εi,T is the regression residual. The apparent activation energy was calculated as bR with R = 8.314462618 J/(mol·K) [22,23,31], then divided by 1000 to express it in kJ/mol.

Residual diagnostics used fitted values calculated from the 33 common-slope Arrhenius input values and the estimated regression coefficients. Formulation effects followed JMP effect coding; the omitted rPP-P50L effect was recovered as the negative sum of the effects for the other ten formulations (0.1064332). Each ordinary residual was calculated as input ln(tR50/h) minus fitted ln(tR50/h). The residual sum of squares (SSE) was 1.315572, and the RMSE with 21 error degrees of freedom was 0.250292.

For the normal Q–Q plot, the 33 ordered residuals were paired with standard-normal quantiles at probabilities (j − 0.5)/33. The reference line was the sample mean residual plus the sample residual SD multiplied by the normal quantile. This SD describes regression-residual scatter, not experimental tensile-test replication.

The common Arrhenius acceleration factor for the 100 °C comparison was calculated using Equation (3). Ea,int is expressed in kJ/mol and multiplied by 1000 to match R in J/(mol·K). The 135 °C anchor and 100 °C target correspond to 408.15 and 373.15 K, respectively.

(3) $${\mathrm{AF}}_{135\to 100}=\exp[\frac{E_{a,\mathrm{int}}\;\times \;1000}{R}\;\times \;(\frac{1}{373.15}-\frac{1}{408.15})]$$

The 100 °C time to 50% retention was obtained by multiplying each formulation’s 135 °C tR50 by the same AF135→100, as in Equation (4).

(4) $$t_{R50,100}\;(h)=t_{R50,135}\;(h)\;\times \;{\mathrm{AF}}_{135\to 100}$$

Hours were converted to years using 365 days per year, or 8760 h/year, as in Equation (5).

(5) $$t_{R50,100}\;(\mathrm{years})=\frac{t_{R50,135}\;(h)\;\times \;{\mathrm{AF}}_{135\to 100}}{8760}$$

The 18 MPa criterion is an absolute-strength endpoint distinct from 50% retention. In JMP’s Destructive Degradation platform, t18 was obtained for each formulation with tensile strength (MPa) as the response, aging time (h) as the time variable, temperature (°C) as the explanatory variable, and a lower specification limit of 18 MPa. A Simple Linear model with linear X/Y scales, a Normal location-parameter path, a common intercept within each formulation, and temperature-specific slopes was used. t18 was defined as the inverse-prediction time at which fitted tensile strength reached 18 MPa. The same Arrhenius acceleration factor was then applied to each formulation’s model-derived t18 at 135 °C, as shown in Equation (6).

(6) $$t_{18,100}\;(\mathrm{years})=\frac{t_{18,135}\;(h)\;\times \;{\mathrm{AF}}_{135\to 100}}{8760}$$

The 18 MPa calculation assumes the transfer of Ea,int from the tR50 analysis; no endpoint-specific activation energy was fitted. The two endpoints are therefore compared conditionally under the same temperature-acceleration assumption. Multiplication by the same positive factor preserves the ranks and paired ratios of the 135 °C inputs and does not experimentally establish the corresponding ordering at 100 °C.

The 33 model-derived tR50 inputs are not independent specimen-level failure times. The three temperatures within a formulation share a fitted degradation model, and the 135 °C anchor is also linked to the inputs used to estimate the common slope. The 95% confidence intervals in Figure 3 describe marginal uncertainty in each inverse-prediction tR50 and are not SDs from experimental replication. They are also not complete confidence intervals for the final tR50,100 or t18,100. Joint propagation of threshold-crossing and activation-energy uncertainty, including their estimation dependence, was not performed; Figure 6 and Figure 7 therefore show point estimates. This distinction separates high-temperature model fit from uncertainty in low-temperature prediction.

The 24 MPa and 75% retention references in GMW16456 were used only to define the 18 MPa research screen [25]. The present endpoint uses the maximum recorded tensile stress and is distinct from conformity testing of tensile strength at yield under the full prescribed procedures. Accordingly, the calculation provides a conditional formulation comparison rather than a formal material qualification.

## 5. Conclusions

The thermal durability and comparative lifetime of talc-reinforced virgin/recycled hybrid PP composites formulated with varying blending ratios of post-consumer recycled PP (PC-rPP) and end-of-life vehicle recycled PP (ELV-rPP) were investigated through accelerated thermal aging at 135, 145, and 155 °C. At fixed contents of 50 wt% recycled feedstock and 20 wt% talc, the tensile and flexural performance generally improved with a decreasing fraction of PC-rPP. The incorporation of LTTS extended $t_{R50}$ relative to the corresponding unstabilized formulations, while the observed initial-property differences did not establish statistical equivalence. Kinetic modeling using an Arrhenius approach with formulation-specific intercepts and a common slope yielded an apparent activation energy ($E_{a,int}$) of 139.86 kJ/mol. Direct extrapolation of the 135 °C reference yielded $t_{R50,100}$ values of 5.11–14.04 years and $t_{18,100}$ values of 2.59–9.14 years. Every LTTS-containing formulation exhibited prolonged threshold times under both criteria. Notably, under the 50% retention criterion, the lifetime of rPP-P10L was comparable to that of vPP, whereas rPP-P20L and rPP-P30L demonstrated even higher values. However, all recycled formulations remained below the performance of vPP under the fixed 18 MPa criterion. These results support the comparative screening of feedstock blends and stabilization conditions. Lowering the total recycled content was not evaluated in this study, and whether such modifications can approach vPP-level durability remains a hypothesis. The 100 °C values are conditional on high-temperature extrapolation and activation energy transfer to the 18 MPa endpoint. Temperature-dependent residuals, the upper-tail Q–Q departure, and the absence of joint uncertainty propagation and direct validation at 100 °C limit their interpretation as definitive service life predictions.

## Figures and Tables

**Table 1:** Initial mechanical properties and DSC melting data of the talc-reinforced virgin and recycled PP composites. Mechanical properties are mean ± sample SD for five specimens per property/test condition. This replicate count and SD do not apply to the DSC melting data.

Sample	Tensile Strength (MPa)	Tensile Modulus (MPa)	Flexural Strength (MPa)	Flexural Modulus (MPa)	Charpy at 23 °C (kJ/m^2^)	Tm (°C)/ΔHm (J/g)
vPP	24.9 ± 0.30	2556 ± 29	35.8 ± 0.31	2314 ± 35	4.4 ± 0.59	165.0/72.0
rPP-P10	23.4 ± 0.32	2458 ± 30	33.0 ± 0.44	2216 ± 35	5.0 ± 0.66	125.2/2.7, 164.2/66.0
rPP-P10L	22.4 ± 0.32	2321 ± 31	31.2 ± 0.42	2014 ± 35	4.9 ± 0.63	125.0/2.8, 164.0/72.7
rPP-P20	21.8 ± 0.28	2445 ± 31	30.8 ± 0.42	2015 ± 36	5.6 ± 0.59	125.7/5.7, 164.4/63.7
rPP-P20L	21.2 ± 0.29	2135 ± 30	29.4 ± 0.38	1813 ± 35	5.5 ± 0.59	125.6/5.9, 164.3/60.6
rPP-P30	21.6 ± 0.29	2242 ± 29	29.4 ± 0.39	1911 ± 36	6.0 ± 0.64	125.9/9.3, 164.1/67.4
rPP-P30L	21.4 ± 0.31	2047 ± 29	28.6 ± 0.35	1811 ± 35	5.6 ± 0.68	126.3/8.9, 164.5/63.7
rPP-P40	21.4 ± 0.29	2214 ± 31	29.1 ± 0.41	1818 ± 35	6.5 ± 0.58	126.5/13.1, 163.8/56.3
rPP-P40L	21.3 ± 0.28	2102 ± 31	28.5 ± 0.43	1818 ± 35	6.4 ± 0.69	126.3/11.4, 164.0/58.7
rPP-P50	20.5 ± 0.29	2181 ± 30	28.7 ± 0.42	1818 ± 35	6.2 ± 0.67	126.7/15.1, 163.8/51.9
rPP-P50L	20.7 ± 0.29	2039 ± 30	27.3 ± 0.34	1613 ± 36	6.9 ± 0.63	126.2/14.6, 164.0/52.1

**Table 2:** Formulation and sample designation of talc-reinforced virgin and recycled PP composites.

Symbol	vPP (wt%)	PC-rPP (wt%)	ELV-rPP (wt%)	Talc (wt%)	Stabilizer (wt%)
vPP	80	0	0	20	0
rPP-P10	30	10	40	20	0
rPP-P10L	29.7	10	40	20	0.3
rPP-P20	30	20	30	20	0
rPP-P20L	29.7	20	30	20	0.3
rPP-P30	30	30	20	20	0
rPP-P30L	29.7	30	20	20	0.3
rPP-P40	30	40	10	20	0
rPP-P40L	29.7	40	10	20	0.3
rPP-P50	30	50	0	20	0
rPP-P50L	29.7	50	0	20	0.3

## Data Availability

The data presented in this study are available from the corresponding author upon reasonable request. The data are not publicly available.
